# Variability of Secondary Metabolites of the Species *Cichorium intybus* L. from Different Habitats

**DOI:** 10.3390/plants6030038

**Published:** 2017-09-11

**Authors:** Nenad M. Zlatić, Milan S. Stanković

**Affiliations:** Department of Biology and Ecology, Faculty of Science, University of Kragujevac, 34000 Kragujevac, Serbia; mstankovic@kg.ac.rs

**Keywords:** saline habitats, secondary metabolites, adaptation, different solvents

## Abstract

The principal aim of this paper is to show the influence of soil characteristics on the quantitative variability of secondary metabolites. Analysis of phenolic content, flavonoid concentrations, and the antioxidant activity was performed using the ethanol and ethyl acetate plant extracts of the species *Cichorium intybus* L. (Asteraceae). The samples were collected from one saline habitat and two non-saline habitats. The values of phenolic content from the samples taken from the saline habitat ranged from 119.83 to 120.83 mg GA/g and from non-saline habitats from 92.44 to 115.10 mg GA/g. The amount of flavonoids in the samples from the saline locality varied between 144.36 and 317.62 mg Ru/g and from non-saline localities between 86.03 and 273.07 mg Ru/g. The IC_50_ values of antioxidant activity in the samples from the saline habitat ranged from 87.64 to 117.73 μg/mL and from 101.44 to 125.76 μg/mL in the samples from non-saline habitats. The results confirmed that soil types represent a significant influence on the quantitative content of secondary metabolites. The greatest concentrations of phenols and flavonoids and the highest level of antioxidant activity were found in the samples from saline soil. This further corroborates the importance of saline soil as an ecological factor, as it is proven to give rise to increased biosynthesis of secondary metabolites and related antioxidant activity.

## 1. Introduction

The abiotic factor with the most harmful effect upon the productivity and growth of plants is soil salinity. Increased concentrations of salt in soil exert a negative influence on plants due to the following reasons: water absorption is more difficult, the growth of the plant may be hindered, and plant metabolism as well as both physiological and chemical processes may be disturbed to a significant extent [1]. In order to successfully adapt to saline soil, plants develop a series of specific mechanisms which enable them to respond appropriately to salinity-induced stress [2]. The mechanisms develop at molecular, cellular, metabolic, and physiological levels [3].

Salinity-induced stress causes the production of reactive oxygen species (ROS), such as hydrogen peroxide, hydroxyl radicals, and superoxide anions in a variety of cells resulting in oxidative stress [4]. Increased production of ROS leads to oxidative damage of cellular membranes, proteins, carbohydrates, and DNA [5]. Plants activate enzymatic and nonenzymatic antioxidant systems for the purpose of protection from the negative influence of free radicals. The activation of enzymatic systems includes superoxide dismutase, catalase, and glutathione reductase, whereas carotenoids, flavonoids, and other phenolic compounds belong to nonenzymatic systems of protection [4]. Different ecological factors that directly influence the plant primary metabolism have different effects. Correspondingly, the differentiation of secondary metabolism occurs. The primary function of secondary metabolites is their participation in the process of plant adaptation to the effects of ecological factors [6]. From an evolutionary viewpoint, biosynthesis of secondary metabolites in plants was considered as an ecophysiological response of a plant to different influences of abiotic factors in a certain habitat, including salinity [7]. Secondary metabolites participate in the process of plant adaptation to the ecological conditions of environment and their quantity in plant organs varies depending on both the abiotic and biotic factors that a plant is exposed to [8].

A great number of investigated medicinal plants that are used in the fields of pharmacy and medicine belong to the family Asteraceae. Within this plant family there are several important genera, which abound in medicinal plant species, and such genera are: *Artemisia*, *Achillea*, *Inula*, and *Matricaria*. These genera show the intense biological activity applied in the treatment of respiratory, digestive, cardiovascular, and some other types of diseases [9]. Apart from therapeutical effects, the active metabolites of the species that belong to this family manifest antimicrobe, antiviral, antiproliferative, and antioxidant activity in both in vitro and in vivo conditions [10,11].

The genus *Cichorium* encompasses approximately nine species. Among the best known medicinal species that belong to the genus *Cichorium* are: *C. intibus*, *C. spinosum*, and *C. dubium*. Chicory (*C. intybus* L., Asteraceae) is a perennial herbaceous plant up to 120 cm high. Its stem is erect and branched. The lower leaves are curled, whereas the upper ones are bare and lanceolate. The flowers are blue in colour and grow either individually or in groups. The fruit is achene, smallish and ovoid in shape. *C. intybus* populates meadows, saline habitats, areas by the road, the edges of forests, and the territories of Europe, West Asia, and North Africa. Due to the limited distribution and wider application, the successful growth of this species is enabled on territories outside its initial area [12].

*Cichorium intybus* is well known in the folk and modern medicine for its anticancer [13], antioxidant [14], antidiabetic [15], anti-inflammatory [16], antimicrobial [17], anthelmintic [18], analgesic [19], cardiovascular [20], gastroprotective [21], hepatoprotective [22], immunological [23], reproductive effects [24], wound healing abilities [25], and many other pharmacological applications [17,26]. It is also important to mention the numerous active substances of this species: alkaloids, coumarins, caffeic acid derivatives, sesquiterpene lactones, flavonoids, terpenoids, and volatile compounds [26].

Secondary metabolites, apart from other adaptive mechanisms, have significance in relation to the reaction of plants to the chemical stress induced by increased quantity of salt in soil in the process of neutralisation of consequences of toxicity for the purpose of ecophysiological adaptation. Taking the previously stated fact into account, the species *C. intybus* was selected as a plant suitable for analysis, with its suitability stemming from the fact that it grows in habitats with both normal mineral regime and with saline soils. Accordingly, by means of sampling the species from one habitat with saline soil and two habitats with normal mineral regime as well as by analysing both the quantity of secondary metabolites from the group of phenolic compounds and their antioxidant activity, a comparison was performed in order to determine their significance in terms of adaptation to toxic effects of salt.

## 2. Results and Discussion

Total phenolic content, flavonoid concentration, and antioxidant activity in vitro were determined using ethanol and ethyl acetate extracts of whole *C. intybus* plant from saline and non-saline habitats. In order to extract the active substances of different polarity, the extraction solvents of different polarity were used.

### 2.1. The Total Quantity of Phenolic Compounds

The results of the analysis of the total quantity of phenolic compounds, flavonoid concentration, and antioxidant activity in ethanol and ethyl acetate extracts of the aboveground plant parts of the species *C. intybus* are shown in Table 1.

The total quantity of phenolic compounds in ethanol extracts ranged from 92.44 to 120.83 mg GA/g of extract, whereas the values in the ethyl acetate extracts varied from 96.55 to 119.83 mg GA/g of extract. The results showed that the greatest quantity of phenolic compounds in the ethanol extracts was found in the sample from the locality Oblačinska slatina (120.83 mg GA/g), while the smallest quantity was observed in the sample collected in Kragujevac (92.44 mg GA/g). Similarly, the highest concentration of phenolic compounds in the ethyl acetate extracts was measured in the samples from the locality Oblačinska slatina (119.83 mg GA/g), whereas the lowest concentration was found in the samples collected in Kragujevac (96.55 mg GA/g). In the extracts obtained using moderate and lower solvents (ethanol and ethyl acetate) the order of concentrations for phenolic compounds is: Oblačinska slatina > Ivanjica > Kragujevac.

The analyses showed that the extract obtained using solvents of lower polarity contained the highest concentration of active compounds. The total quantity of phenolic compounds was greater in the ethanol extracts made from the solvent of moderate polarity and the samples collected from the locality of Oblačinska slatina (120.83 mg GA/g of extract). Consequently, it was shown that a certain group of phenolic compounds is important for the adaptation to saline habitats [27].

The total quantity of phenolic compounds in the aboveground plant parts of the species *C. intybus* varied depending on the type of locality from which the plant material was taken. The substrate of Oblačinska slatina contains high concentrations of salt. Contrary to this, the substrates of Ivanjica and Kragujevac contain low salt concentrations. Due to this difference in salt concentrations, greater quantities of phenolic compounds (120.83 mg GA/g) were measured in the plant extract of the sample from Oblačinska slatina. The obtained results for the quantity of phenolic compounds of the species *C. intibus* are in accordance with the previously recorded values [28]. It has been confirmed that the quantity of polyphenols in plants belonging to the species *Mentha pulegium* increases due to the stress caused by greater concentrations of salt in ground substrate [29]. Similarly, the examination of the species *Nigella sativa* demonstrated the increase in the total quantity of phenols due to saline treatment [30]. Salinity-induced increases in the concentration of the group of phenolic acids (protocatechuic, chlorogenic, and caffeic acids) has been observed in the species *Matricaria chamomilla* [31].

The plants sampled in Ivanjica contained higher concentrations of phenolic compounds in comparison with the samples from Kragujevac. These localities differ in altitude, as Ivanjica is located at an altitude of 997 m and Kragujevac at an altitude of 194 m. Previously performed studies showed that the plant species in habitats with increased intensity of light contain phenolic compounds, which have considerable influence on the adaptational abilities of plants populating these habitats. The increased quantity of phenolic compounds in plants plays a protective role from ultraviolet-B radiation, which is more intense at higher altitudes [32,33].

### 2.2. The Total Quantity of Flavonoids

The results of the analysis of the total quantity of flavonoids in ethanol and ethyl acetate extracts of aboveground plant parts of the species *C. intybus* are shown in Table 1. The total quantity of flavonoids in the ethanol extracts ranged from 86.03 to 144.36 mg Ru/g of extract, whereas the quantity in the ethyl acetate extracts varied from 176.09 to 317.62 mg Ru/g of extract. The results showed that the greatest quantity of flavonoids in the ethanol extracts was found in the sample from the locality Oblačinska slatina (144.36 mg Ru/g), while the smallest quantity was observed in the sample collected in Kragujevac (86.03 mg Ru/g). Similarly, the highest concentration of flavonoids in the ethyl acetate extracts was measured in the sample from the locality Oblačinska slatina (317.62 mg Ru/g), whereas the lowest concentration was found in the sample collected in Kragujevac (176.09 mg Ru/g). In the extracts obtained using moderate and lower solvents (ethanol and ethyl acetate) the order of concentrations for phenolic compounds is: Oblačinska slatina > Ivanjica > Kragujevac.

The concentration of flavonoids in plant extracts depends on the polarity of solvents used in the extract preparation. Based on the obtained values for concentration of flavonoids, the highest concentration of these compounds is observed in the extracts obtained using solvents of low polarity. High concentrations of flavonoids in ethyl acetate extracts may be attributed to their high solubility in this solvent [34]. Accordingly, the analysis of whole plant extracts indicated that ethyl acetate was the most effective solvent for the extraction of flavonoids from the species *C. intybus* and that therefore solvents with low and moderate polarity should be used to this end.

Apart from solvent polarity, what proved to be relevant in terms of the quantity of flavonoids is the type of locality from which samples were taken. The greater quantity of flavonoids was observed in the extract made from the samples collected from the locality of Oblačinska slatina (317.62 mg Ru/g) the substrate of which contains high concentrations of salt. Previous studies confirmed that certain plants from saline habitats synthetize greater concentrations of flavonoids as a response of the secondary metabolism to the adaptation of species to the increased level of salt in the substrate [35,36].

The concentration of flavonoids differs in the localities of Ivanjica and Kragujevac, with Ivanjica having higher concentrations of flavonoids in both types of extracts. This study revealed a difference in the quantity of flavonoids in the samples collected at different altitudes. Increases in altitude may cause increases in the plant production of flavonoids. UV-B radiation increases the production of flavonoids in barley [37]. The greater intensity of light in a habitat is accompanied by a larger accumulation of secondary metabolites in certain plant organs [32].

### 2.3. Antioxidant Activity

The obtained values for antioxidant activity of the ethanol and ethyl acetate extracts made from the aboveground plant parts of the species *C. intybus* are shown in Table 1. Antioxidant activity of the ethanol extracts from different localities ranged from 117.73 to 121.05 μg/mL, while the antioxidant activity of the ethyl acetate extracts varied from 87.64 to 125.76 μg/mL. The results showed that the highest level of antioxidant activity of both the ethanol and ethyl acetate extracts was observed in the samples from the locality Oblačinska slatina, with values of 87.64 μg/mL and 117.73 μg/mL for each type of extract, respectively. The lowest values of both the ethanol and ethyl acetate extracts were obtained from the samples collected in Kragujevac, with values of 121.05 μg/mL and 125.76 μg/mL for each type of extract, respectively. In the extracts obtained using moderate and lower solvents (ethanol and ethyl acetate) the order of antioxidant activity is: Oblačinska slatina > Ivanjica > Kragujevac.

The extraction of antioxidant substances of different chemical structure was achieved using solvents of different polarity. The largest capacity to neutralize DPPH (2,2-dyphenyl-1-picrylhydrazyl) radicals was measured in the ethyl acetate extract from the saline habitat Oblačinska slatina, which neutralized 50% of free radicals at concentrations of 87.64 µg/mL. It is well known that the phenolic content of plants may contribute directly to their antioxidant activity [34] due to its role in scavenging free radicals.

The plants sampled from the locality Oblačinska slatina had the highest concentrations of phenols and flavonoids. This indicates that secondary metabolites of the phenolics group in *C. intybus* are the key active substances for the expression of antioxidant activity. Earlier studies on major secondary metabolites in upper parts of *C. intybus* demonstrated the presence of sesquiterpene lactones as well as caffeic acids derivates (chicoric acid, chlorogenic acid, isochlorogenic acid, dicaffeoyl tartaric acid). Significant biological activity of these phenol acids and flavonoids has already been confirmed for both in vitro and in vivo model systems. The antioxidant activity has been reported as well [26].

Numerous studies have shown increased activity of antioxidant enzymes in plants located in habitats with saline substrates. Neutralisation of free radicals in the species *Phragmites karka* increases when the plant is exposed to higher concentrations of salt [38]. Similar results have been obtained for the species *Hordeum vulgare* [39].

In comparison with the locality of Kragujevac, Ivanjica stands out in terms of its greater ability to neutralise free radicals. This is attributable to the differences in the type of habitat and altitude. These results are in accordance with the findings that the extracts of the samples collected at higher altitudes have greater antioxidant capacity than the extracts of the samples taken at lower altitudes [40,41].

### 2.4. Correlation between Phenolic Compounds, Flavonoids, and Antioxidant Activity

The obtained results for the total quantity of phenolic compounds and flavonoids, as well as for the level of antioxidant activity of the ethanol and ethyl acetate extracts of the species *C. intybus* were statistically analysed for the purpose of determining the degree of correlation. The values of correlation point to the significant link between the analysed parameters.

The correlation between the total quantity of phenolic compounds, flavonoids, and the values of antioxidant activity measured in both ethanol and ethyl acetate extracts is established in *C. intybus* (Table 2). It can be noticed that an increase in the phenol content of extract decreases the IC_50_ value, i.e., increases their scavenging DPPH free-radical activity (negative correlation). This is important in the light of the fact that the lower IC_50_ values imply higher levels of antioxidant activity. Similar conclusions have been reached in other studies [42,43].

## 3. Materials and Methods

### 3.1. Plant Material

Plants were sampled from natural populations found in different localities with saline and non-saline substrate. The plant material was sampled from three different localities: one saline (Oblačinska slatina, Serbia) and two non-saline ones (Ivanjica and Kragujevac, Serbia). The sampling was performed in the phase of flowering in August 2014 (Table 3). The collected samples were identified in the Department of Biology and Ecology of the Faculty of Science in Kragujevac. Aboveground plant parts were dried in a dark room at room temperature. Dry samples were ground in a blender and thus kept in dark vials until analysis.

### 3.2. Preparation of Plant Extracts

Dried plant material (10 g) was put into Erlenmeyer flasks filled with 200 mL of ethanol or ethyl acetate and thus left at room temperature. The extract was filtrated after 48 h using Whatman No. 1 filter paper. The plant extracts were condensed to dry on a rotary vacuum at 40 °C. The obtained extracts were placed in sterile containers and kept in the fridge at 4 °C. The total quantity of phenolic compounds, flavonoids, as well as the level of antioxidant activity were determined in the extract concentration of 1 mg/mL using ethanol or ethyl acetate as solvents.

### 3.3. Chemicals

Organic solvents and sodium hydrogen carbonate were purchased from Zorka pharma Sabac, Serbia. 2,2-dyphenyl-1-picrylhydrazyl (DPPH) was obtained from Sigma Chemicals Co., St. Louis, MO, USA. Folin-Ciocalteu phenol reagent, 3-tert-butyl-4-hydroxyanisole (BHA), and aluminium chloride hexahydrate (AlCl_3_·6H_2_O) were purchased from Fluka Chemie AG, Buchs, Switzerland. All other solvents and chemicals were of analytical grade.

### 3.4. Determination of Total Phenolic Contents

The total phenolic content was determined using the spectrophotometric method [44]. First, 0.5 mL of methanol solution (1 mg/mL) of extract, 2.5 mL of 10% Folin-Ciocalteu’s reagent dissolved in water, and 2.5 mL 7.5% NaHCO_3_ were used to obtain the reaction mixture. Then, the samples were incubated at 45 °C for 15 min. The absorbance was measured at λ_max_ = 765 nm. The samples were prepared in triplicate and the mean value of absorbance was obtained. The blank was prepared with methanol solution. The same procedure was repeated for the gallic acid and the calibration curve was constructed. The total phenolic content was expressed as gallic acid equivalent (mg of GA/g).

### 3.5. Determination of Flavonoid Concentrations

The concentration of flavonoids was determined using the spectrophotometric method [45]. The sample contained 1 mL of methanol solution of the extract in the concentration of 1 mg/mL and 1 mL of 2% AlCl_3_ solution dissolved in methanol. The samples were incubated at ambient temperature for an hour. The absorbance was measured at λ_max_ = 415 nm. The samples were prepared in triplicate and the mean value of absorbance was obtained. The same procedure was repeated for the rutin and the calibration line was constructed. Concentration of flavonoids in extracts was expressed in terms of rutin equivalent (mg of Ru/g).

### 3.6. Evaluation of DPPH Scavenging Activity

The efficiency of the plant extract to neutralise DPPH (1,1-diphenyl-2-picrylhydrazyl radical) free radicals was determined using the spectrophotometric method previously described [46] with adequate modifications [47]. The plant extract was dissolved in methanol to obtain the concentration 1 mg/mL. Dilutions were made to obtain concentrations of 500, 250, 125, 62.5, 31.25, 15.62, 7.81, 3.90, 1.99, and 0.97 mg/mL. Diluted solutions (1 mL each) were mixed with 1 mL of DPPH methanolic solution (80 mg/mL). The absorbance was recorded at 517 nm. The control samples contained methanol and DPPH reagents. The percentage inhibition was calculated using the equation: % inhibition = 100 × (A of control − A of sample)/A of control)), whilst IC_50_ values were estimated based on the sigmoidal curve presenting the dependence of the percent of DPPH scavenging on sample concentration. Antioxidant activity was expressed as the half-maximal inhibitory concentration (IC_50_ values in mg/mL). In the presented results, antioxidant efficiency of the extract increased with decreasing IC_50_ values. The data were presented as mean values ± standard deviation (*n* = 3).

### 3.7. Statistical Analysis

All experimental measurements were carried out in triplicate and are expressed as the average of three analyses ± standard deviation. Results were analysed statistically using IBM, SPSS, Statistics, ver. 19, Armonk, NY: IBM Corp. Pearson's correlation coefficient (r) was used to evaluate relationships between contents and antioxidant properties of chicory extracts.

## 4. Conclusions

The results of this research show that there is a significant difference in the quantity of secondary metabolites and their activity in the species *C. intybus*, which populates both saline and non-saline habitats. The total quantity of phenolic compounds and flavonoids increases due to the presence of salt in the substrate. The level of antioxidant activity was higher in the samples taken from saline habitats, and the result implies that this is a mechanism of plant adaptation to the increased concentrations of salt in the substrate. Plants may adapt to the stressful conditions in the habitat by means of synthesis regulation and accumulation of secondary metabolites. The plants tolerant to salt stress are sources of secondary metabolites and may be highly applicable in pharmaceutical and food industries.

## Figures and Tables

**Table 1:** Total phenolic content, flavonoid concentration, and antioxidant activity of the analysed species in ethanol and ethyl acetate extracts.

Locality	Type of Analysis					
	Total Phenolic Content (mg of GA/g of Extract)		Flavonoid Content (mg of Ru/g of Extract)		Antioxidant Activity IC_50_ (µg/mL)	
	Type of Extract					
	Ethanol	Ethyl Acetate	Ethanol	Ethyl Acetate	Ethanol	Ethyl Acetate
Oblačinska slatina	120.83 ± 1.02	119.83 ± 1.34	144.36 ± 0.83	317.62 ± 2.04	117.73 ± 1.71	87.64 ± 1.90
Ivanjica	95.53 ± 0.97	115.10 ± 1.50	129.00 ± 1.18	273.07 ± 1.56	120.53 ± 2.21	101.44 ± 1.53
Kragujevac	92.44 ± 1.12	96.55 ± 1.45	86.03 ± 0.59	176.09 ± 1.39	121.05 ± 1.66	125.76 ± 2.33

Each value is the average of three analyses ± standard deviation.

**Table 2:** The coefficient of correlation (*p* < 0.05) between the total phenolic compounds (TPC), total flavonoids (TF) and the values for antioxidant activity (AA).

(r)	AA Ethanol	AA Ethyl Acetate
TPC Ethanol	−0.999 *	−0.835
TPC Ethyl acetate	−0.760	−0.985
TF Ethanol	−0.800	−0.994
TF Ethyl acetate	−0.832	−0.999 *

* Correlation is significant at the 0.05 level.

**Table 3:** The basic characteristics of the localities in which the species *C. intybus* was sampled.

Locality	Type of Habitat	Altitude	Latitude and Longitude
Oblačinska slatina	Meadow, Hygrophilous habitat	285 m	43°18′17.76″ N 21°41′0.340″ E
Ivanjica	Meadow, Mesophilous habitat	997 m	43°28′32.55″ N 20°10′29.11″ E
Kragujevac	Meadow, Mesophilous habitat	194 m	44°01′31.18″ N 20°54′50.62″ E
